# Determination of Background Concentrations of Ag, Pd, Pt and Au in Highly Mineralized Ground Waters at Sub-ng L^–1^ Concentrations by Online Matrix Separation/Pre-Concentration Coupled to ICP-SFMS

**DOI:** 10.3390/molecules26237253

**Published:** 2021-11-29

**Authors:** Lisa Fischer, Bernadette Moser, Stephan Hann

**Affiliations:** Department of Chemistry, University of Natural Resources and Life Sciences, BOKU Vienna, 18 Muthgasse, 1190 Vienna, Austria; bernadette.moser@boku.ac.at (B.M.); stephan.hann@boku.ac.at (S.H.)

**Keywords:** trace metals, ground water, ICP-SFMS, strong anion exchange resin, isotope dilution analysis

## Abstract

Though not regulated in directives such as the Water Framework Directive of the European Union, the investigation of geogenic background concentrations of certain elements such as precious metals is of increasing interest, in particular for the early detection of a potential environmental pollution due to the increased use in various industrial and technological applications and in medicine. However, the precise and accurate quantification of precious metals in natural waters is challenging due to the complex matrices and the ultra-low concentrations in the (sub-) ng L^−1^ range. A methodological approach, based on matrix separation and pre-concentration on the strong anion exchange resin TEVA^®^ Resin in an online mode directly coupled to ICP-SFMS, has been developed for the determination of Ag, Pt, Pd and Au in ground water. Membrane desolvation sample introduction was used to reduce oxide-based spectral interferences, which complicate the quantification of these metals with high accuracy. To overcome errors arising from matrix effects—in particular, the highly varying major ion composition of the investigated ground water samples—an isotope dilution analysis and quantification based on standard additions, respectively, were performed. The method allowed to process four samples per hour in a fully automated mode. With a sample volume of only 8 mL, enrichment factors of 6–9 could be achieved, yielding detection limits <1 ng L^−1^. Validation of the trueness was performed based on the reference samples. This method has been used for the analysis of the total concentrations of Ag, Pt, Pd and Au in highly mineralized ground waters collected from springs located in important geological fault zones of Austria’s territory. Concentrations ranges of 0.21–64.2 ng L^−1^ for Ag, 0.65–6.26 ng L^−1^ for Pd, 0.07–1.55 ng L^−1^ for Pt and 0.26–1.95 ng L^−1^ for Au were found.

## 1. Introduction

Natural mineral waters and medicinal waters are certain types of ground waters that are distinguished from drinking water by their high purity at the source and specific chemical and physical characteristics. Their beneficial impact on the human organism has been well-known for thousands of years. The type of occurrence and the level of minerals primarily depend on the bed rock geochemistry. Directive 2009/54/EC [1] regulates the exploitation and marketing of natural mineral water, and in accordance with that, the European Commission published and regularly updates a list of natural minerals waters that are officially recognized by the EU countries in the official *Journal of the European Union* [2].

The protection of these valuable water sources is of the highest priority. Ground water reserves in deeper basins are naturally protected by multilayered rock formations against pollutant inputs to a greater extent than shallow ground waters in karst and crevice rock formations. Percolating rain water, artificially exploited water sources through bore holes and diverse human activates may endanger this natural protection. Apart from the “conventional nonsynthetic” pollutants such as As, Cd, Hg and Pb, etc. regulated in the Water Framework Directive (Directive 2000/60/EC) [3] and the Ground Water Directive (Directive 2006/118/EC) [4], there is an increasing interest in knowledge about the occurrence and (natural) concentrations of metals critical to emerging technologies—the so-called technology-critical elements [5]. In particular, the interest in the determination of precious metal concentrations in natural waters is steadily increasing because of potential anthropogenic emissions resulting from their use in a variety of modern industrial, chemical, electrical, pharmaceutical and medical applications. Pt and Pd (and Rh) have become very prominent since their use as catalytic converters for cars. Some studies have already shown the potential risks of some precious metals to human health and ecology [6,7].

The determination of (background) concentrations of these elements in natural waters is still in its infancy, not least because of the naturally occurring concentrations in the sub-ng L^−1^ to pg L^−1^ range, which require very sensitive instrumentation in combination with powerful extraction and pre-concentration methods. The most commonly applied atomic spectrometric techniques for the analysis of Ag, Pd, Pt and Au (mostly in saline waters) are graphite furnace/electrothermal atomic absorption spectrometry (GF-AAS/ET-AAS) [8,9,10,11,12] and inductively coupled plasma (sector field) mass spectrometry (ICP-(SF)MS) [13,14,15,16,17,18,19,20,21,22]. By now, solid-phase extraction (SPE) techniques on chelating sorbent materials prior to detection in either an off-line mode or directly coupled to the detection system are replacing liquid–liquid extraction methods using ammonium 1-pyrrolidinedithio-carbamate/diethylammonium diethyldithiocarbamate (APDC/DDDC), as (first) described by Bruland and Franks [23] for the pre-concentration of Cu, Cd, Zn and Ni and co-precipitation and flotation techniques.

Since Ag, Pd, Pt and Au mainly occur as anionic chloro complexes AgCl_2_^−^ and AgCl_3_^2−^; PdCl_4_^2−^ and PdCl_3_OH^2−^; PtCl_4_^2−^ and PtCl_3_(OH)^2−^ and AuCl_2_^−^, AuCl_3_(OH)^−^, AuCl_2_(OH)^2−^ and AuCl(OH)^3−^ in the aqueous phase [24], facilitated by the chloride content in natural waters, SPE based on (strong) anion exchangers is the method of choice. For the extraction of Ag from estuaries and seawater, SPE procedures using silica-immobilized 8-hydroxyquinoline [25], chelating ion exchange resins [22] (Chelex-100; styrene divinylbenzene copolymers containing paired iminodiacetate ions) and strong anion exchangers (Dowex^®^ AG 1-X; styrene-divinylbenzene-immobilized trimethylbenzylammonium functional groups) [13,14,15,18] were applied in batch or continuous extraction mode. SPE procedures based on polyaniline [17] and 1.5-bis(2-pyridyl)-3-sulphophenyl methylene thiocarbonohydrazide immobilized on aminopropyl-controlled pore glass (PSTH-cpg) [16] were used for the extraction of Pd from ground-, lake- and seawater in an online mode directly coupled to ICP-MS. The latter method [16] was also applied to the analysis of Pt (and Ir) in spiked seawater and river water samples. The Dowex^®^ AG 1-X resin has also been used for the extraction of Pt from seawater prior to isotope dilution ICP-MS [19,20]. Techniques for the analysis of Au from seawater include SPE on Amberlite XAD-2000 [12] (polystyrene divinylbenzene copolymer) prior to F-AAS and the extraction of Au on AG 1-X2 as the cyanide complex (Au(CN)_2_^−^) following FI-ICP-MS [21]. A more detailed description and discussion of, in particular, online sample preparation coupled to atomic detection for the determination of trace elements (including precious metals) in natural waters can be found elsewhere [26]. The accurate determination of precious metals is also hampered by numerous spectral interferences, mainly oxide-based interferences such as ZrO^+^, ZrOH^+^ and MoOH^+^ on the Ag isotopes; MoO^+^, SrO^+^ and SrOH^+^ on the Pd isotopes and HfO^+^ interferences on all Pt isotopes. In addition to that, isobaric interferences such as Cd interferences on ^106^Pd and ^108^Pd or Hg interferences on ^196^Pt and ^198^Pt, which are not resolvable even by a mass resolution of *m/*∆*m* < 10,000, complicate the analysis.

**Figure 1:**
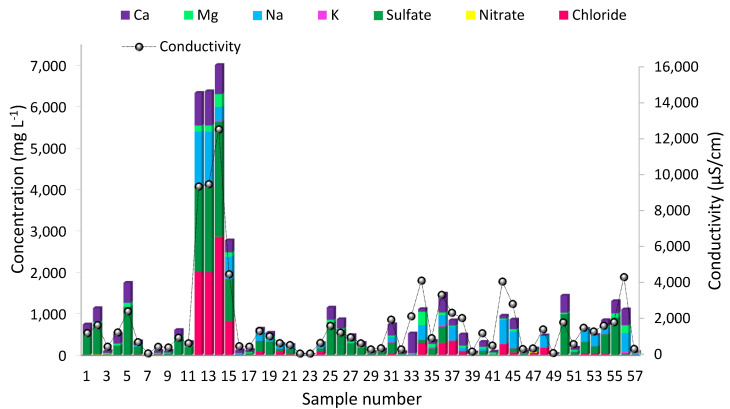
Average major ion composition and conductivity of the investigated ground water samples.

In this work, we report the development and optimization of a methodological approach for the determination of selected precious metals (Ag, Pd, Pt and Au) in highly mineralized ground waters, also referred to as mineral waters according to the regulations, based on SPE on the strong anion exchange resin TEVA^®^ Resin and online coupled to ICP-SFMS using membrane desolvation as a sample introduction system. This study was performed within a project in cooperation with the Federal Ministry of Federal Ministry of Agriculture, Regions and Tourism of the Republic of Austria and the Environmental Agency of Austria. The overall scope of the project was the characterization of 55 springs located in the most important geological fault zones of Austria’s territory, which have not yet been investigated with respect to their elemental compositions. In total, 66 elements, including precious metals, rare earth elements and Hg, were investigated (the results were published in Reference [27], German version only). The average major ion composition of the investigated samples (shown in Figure 1) clearly indicates very diverse sample matrices and partly high and varying degrees of mineralization. Due to this, the direct analysis without dilution of the samples was prone to matrix suppression and signal drift, leading to incorrect results; on the other hand, further dilution of the analytes in the sample resulted in concentrations below the quantification limit. This was remarkable for a suite of precious metals; hence, more advanced methods for their analysis were required.

## 2. Results and Discussion

### 2.1. Optimization of the Procedure for Matrix Separation and Pre-Concentration

Continued development of a methodology for the quantification of Ag, Pd, Pt and Au at ng L^−1^ and sub-ng L^−1^ concentrations, respectively, in higher mineralized natural waters, which cannot be measured directly/undiluted by ICP-MS, was based on an off-line matrix separation/pre-concentration method for the analysis of dissolved Ag and Pt in seawater [28]. In brief, in this method, a four-channel manifold with pre-concentration columns filled with the strong anion exchanger Dowex^®^ 1x8 was used to manually process volumes of each 100 g of seawater acidified with 0.024-mol L^−1^ HCl. Elution with 2.5 g of a mixture of 4-mol L^−1^ HNO_3_ + 0.5-mol L^−1^ HCl led to enrichment factors of 40–45, which allowed an open ocean seawater analysis.

The present study aimed at automation and the operation of the automated system in an online mode, hence direct coupling to the ICP-SFMS for transient signal data acquisition. Compared to the off-line matrix separation/pre-concentration manifold, <10 mL of sample volume was loaded onto the column; however, the flow rates for sample loading, rinsing and pre-conditioning remained at 2 mL min^−1^ (see Section 3.3.1).

As more and novel anion exchanger resins, respectively, have been available recently, these were also tested for extraction of the selected metals—in particular, the TK201 resin B (tertiary amine), TEVA^®^ Resin (aliphatic quaternary amine), DGA resin (*N,N,N’,N’*-tetra-2-ethylhexyldiglycolamide) and the CL resin. Aliquots of diluted (1:10) seawater samples were spiked with 10 ng L^−1^ of Ag, Pd, Pt and Au and extracted under identical conditions on the different resins. A comparison of the elution profiles showed a low signal response and wide and tailing peaks when using the DGA and CL resins (results are not shown), indicating a weaker affinity for the target analytes and/or a very strong retention and weak elution with the used acid solution.

**Figure 2:**
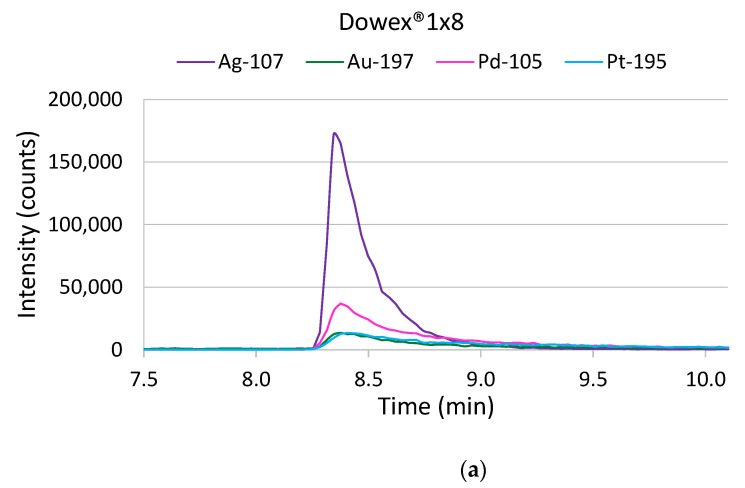

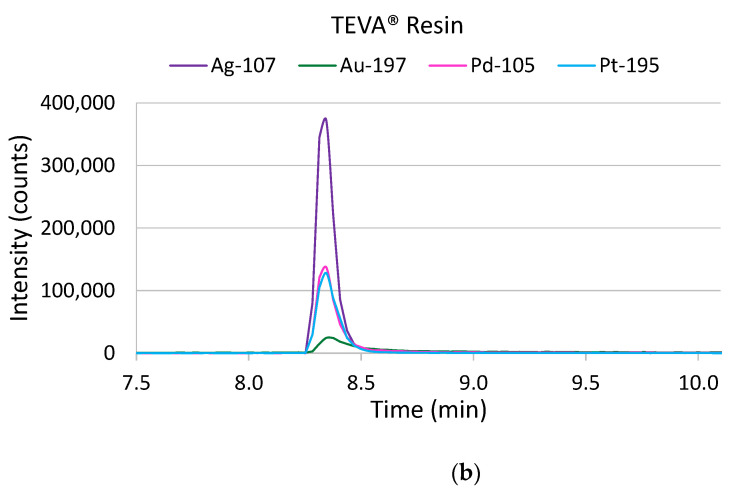
Elution profiles obtained from matrix separation/pre-concentration of seawater spiked with 10 ng L^−1^ of Ag, Pd, Pt and Au on the strong anion exchangers Dowex^®^1x8 (**a**) and TEVA^®^ Resin (**b**).

In contrast to Dowex^®^1x8, the signal responses strongly increased after extraction of the target metals on the TEVA^®^ Resin, and the elution profiles showed higher and narrower symmetric peak shapes compared to those after extraction on Dowex^®^1x8. Figure 2 shows the elution profiles obtained for Ag, Pd, Pt and Au (10 ng L^−1^) in 10% seawater. Additionally, TK201 resin B was tested, and similar characteristics with respect to the signal response and peak width were obtained (elution profile not shown). The only differences between TEVA^®^ resin and TK201 resin B were the peak shapes of the Pd isotopes, which showed a slight tailing when using TK201 resin B. Hence, the TEVA^®^ resin was chosen for further experiments to optimize the matrix separation and pre-concentration procedures for the quantification of Ag, Pd, Pt and Au in ground water samples.

Initial experiments were performed to study the effects of the column length and the sample volume loaded onto the column. Therefore, PEEK columns of different sizes (50 × 2.1-mm inner diameter; volume ≈ 173 µL and 30 × 2.1-mm inner diameter; volume ≈ 104 µL) packed with resin were used. Seawater samples (1:10 diluted) spiked with 10 ng L^−1^ of a standard containing Ag, Pd, Pt and Au were analyzed with both columns. Signal intensities of eluted interfering matrix components and Sr and Mo significantly decreased when the smaller pre-concentration column (104-µL volume) was used, whereas those of the target elements increased by a factor of two. Signal intensities resulting from sample volumes of 4 mL and 8 mL were compared, and the highest intensities were obtained when 8 mL of sample were loaded on the smaller column; hence, good matrix separation and pre-concentration of the target analytes could be achieved.

A series of experiments was further conducted, including parameters such as sample acidity, composition and acidity of elution reagents and matrix effects.

**Figure 3:**
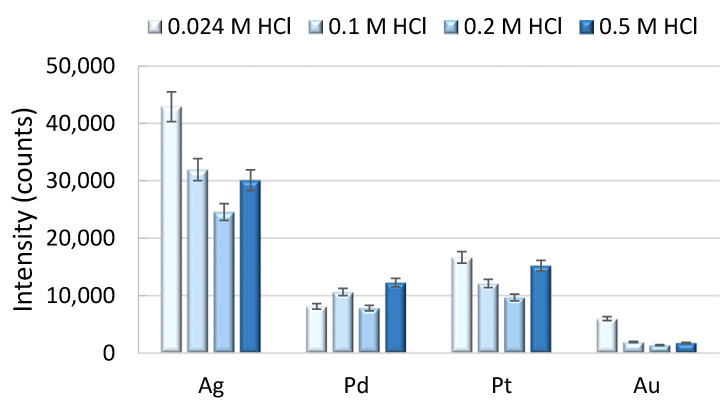
Effect of the chloride concentration in the sample on the retention of the target analytes (1:10 diluted seawater samples were spiked with 10 ng L^−1^ of the target elements). Error bars represent the estimated measurement uncertainty (2 s) calculated from replicate measurements of a seawater standard spiked with 10 ng L^−1^ of Ag, Pd, Pt and Au (*n* = 3).

The chloride concentration in water is one of the main factors influencing the speciation of the target metals and, hence, retention of the respective chloro complexes with the anion exchange resin. The modeling of inorganic Ag speciation, as performed by Barriada et al. [29], showed an increase of double and triple negatively charged Ag chloro complexes (AgCl_3_^2−^, AgCl_4_^3−^) at increased salinities (chloride concentrations ranging from 0 to 0.55 mol L^−1^ representing low-salinity riverine and estuarine waters and seawater) and, hence, increased Ag signal response with increasing salinity after the extraction of Ag from seawater on the strong anion exchanger Dowex^®^ 1x8. This effect was also investigated by another study [13], whereby sample solutions with different NaCl concentrations (0–10% NaCl and seawater spiked with Ag) were adjusted with varying concentrations of HCl (0–2 mol L^−1^). The authors reported the most efficient Ag retention at chloride concentrations from 0.05 to 0.5 mol L^−1^ and decreased retention at lower or higher total chloride concentrations as a result of incomplete Ag chloro complex formation and an excess of chloride in the sample, respectively. We studied this effect by extracting aliquots of 10% (*v/v*) seawater spiked with 10 ng L^−1^ of Ag, Pd, Pt and Au with HCl at concentrations ranging from 0.024 to 0.200 mol L^−1^ on the TEVA^®^ Resin. The results of this experiment are shown in Figure 3. The highest signal response for Ag and Au could be obtained with a HCl concentration of 0.024 mol L^−1^ in the sample, whereas the effect was most significant for the response of Au, as the signal intensity was increased by 70% compared to these obtained with higher HCl concentrations. Though the speciation of gold in the aqueous environment is very diverse [24], the Au chloro complexes Au(III)Cl_4_^−^ and Au(I)/(III)Cl_2_^−^ are considered to be the dominant species in oceanic waters, but the AuOH(H_2_O) complex is also present in oxygenated ocean waters [30], and consequently, it can be assumed that the chloro complex formation is driven by the chloride content in the sample. The response for Pd and Pt was not affected by this extent, as the signal intensities obtained at different acidities were in the range of ±20%.

**Figure 4:**
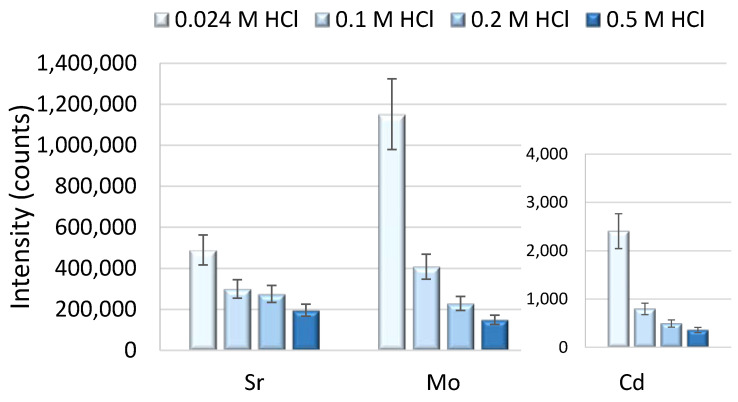
Effect of the chloride concentration in the sample on the retention of the interfering analytes: Sr, Mo and Cd.

Furthermore, it could be shown that the signal response for Mo and Sr, whose oxides and hydroxides form polyatomic interferences on the Ag^−^ and Pd^−^ isotopes, and Cd, which is isobarically interfering on the Pd isotopes ^106^Pd, ^108^Pd and ^110^Pd, decreased with increasing HCl concentrations in the sample (see Figure 4). This finding has been also published earlier for Mo, Zr and Nb [13], and thus, the researchers chose a higher HCl concentration to minimize polyatomic interferences on the Ag isotopes. Our results showed that an increase in the HCl concentration from 0.024 mol L^−1^ to 0.1 mol L^−1^ resulted in a decrease of the interfering species of approximately 65% and further decreased with further increasing the HCl concentration in the sample solution. However, aiming at the highest possible response for Au, an HCl concentration of 0.024 mol L^−1^ was chosen, and the reduction of oxide-based interferences by using membrane desolvation sample introduction as an alternative to a conventional glass or Teflon^®^ PFA spray chamber was applied (see Section 2.2).

**Figure 5:**
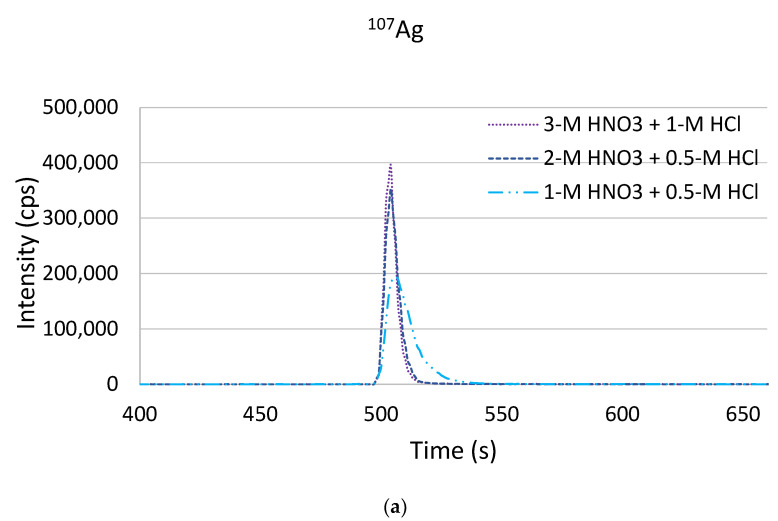

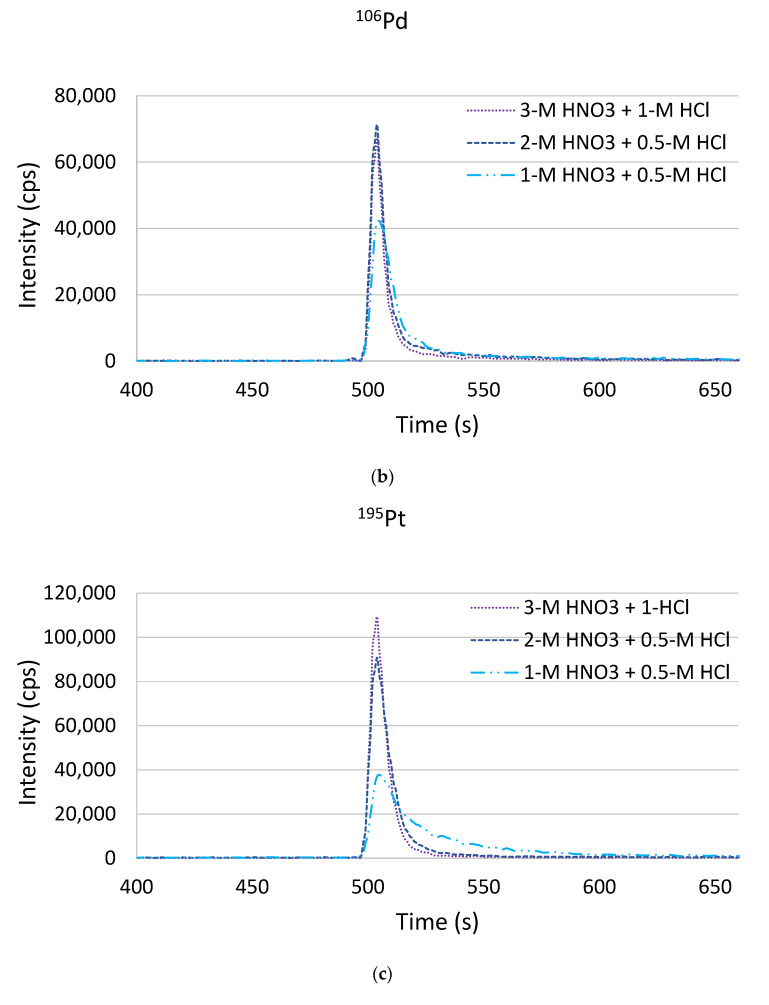

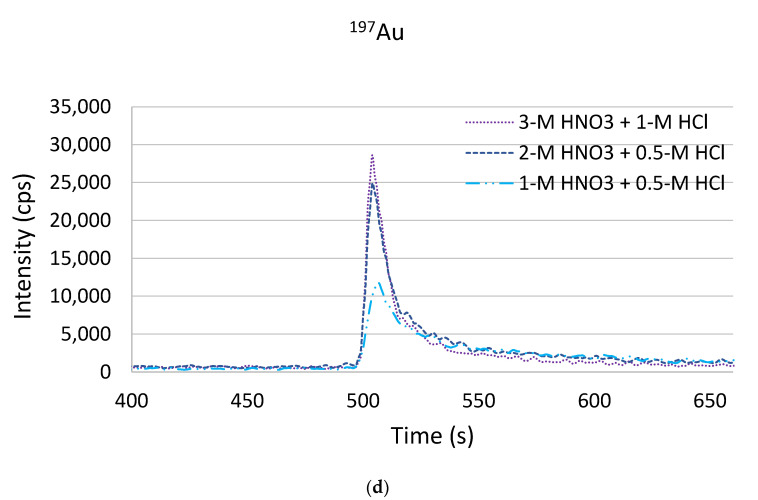
Effects of different elution acid strengths and compositions on the elution of Ag (**a**), Pd (**b**), Pt (**c**) and Au (**d**) (10 ng L^−1^ in seawater) from TEVA^®^ Resin.

The elution conditions were optimized with respect to the acid strength and composition. Previous experiments were performed with an elution acid containing 4-mol L^−1^ HNO_3_ + 1-mol L^−1^ HCl. The eluted metals extracted on the TEVA^®^ Resin showed high and narrow peaks, with a peak width of only 25–30 s compared to 55–110 s when Dowex^®^ 1x8 was used (see Figure 2). Hence, we tested weather weaker elution acids also led to an effective elution. Seawater was spiked with 10 ng L^−1^ of Ag, Pd, Pt and Au, and for the elution, three different eluent solutions were tested: 3-mol L^−1^ HNO_3_ + 1-mol L^−1^ HCl, 2-mol L^−1^ HNO_3_ + 0.5-mol L^−1^ HCl and 1-mol L^−1^ HNO_3_ + 0.5-mol L^−1^ HCl. The obtained elution profiles are shown in Figure 5. It could be seen that the eluent composition with the lowest acid strengths resulted in significantly lower signal intensities and broadening of the peaks. This could be observed for all the target elements to a similar extent. However, the use of an elution acid mixture of 3-mol L^−1^ HNO_3_ + 1-mol L^−1^ HCl or 2-mol L^−1^ HNO_3_ + 0.5-mol L^−1^ HCl, respectively, did not show significant differences compared to the initially used 4-mol L^−1^ HNO_3_ + 1 mol L^−1^. As a result, a mixture of 2-mol L^−1^ HNO_3_ + 0.5-mol L^−1^ HCl was chosen for the elution of the target analytes.

To conclude, the extraction of Ag, Pd, Pt and Au on the TEVA^®^ Resin showed advantages with respect to the signal response, the peak width and the shape and the elution conditions. Table 1 shows a comparison of the sensitivities obtained estimated from standard addition curves in 1:10 diluted seawater (concentrations ranged from 0 to 10 ng L^−1^). It can be clearly seen that the sensitivities increased by a factor of approximately two (Au) to five (Ag) when using the strong anion exchange resin TEVA^®^ Resin. While the peak tailing was severe when using Dowex^®^1x8, resulting in broad signals with 55 s (Ag) to 110 s (Pt, Pd and Au), narrow and symmetrical peaks only 25–30-s wide were obtained for Ag, Pd and Pt with the TEVA^®^ Resin; hence, theoretical pre-concentration factors of approximately 9–16 could be obtained from a sample volume of 8 mL. It has to be pointed out that, for elution on TEVA^®^ Resin, only half the acid strength, compared to Dowex^®^1x8, was required for an efficient elution of the target analytes. The elution time was set to 5 min at a flow rate of 1 mL min^−1^ to sufficiently clean the resin to prevent carryover. In a follow-up project, where this method was applied for the analysis of Ag in seawater, the eluent flow rate was reduced to 400 µL min^−1^ without sacrificing the performance, which made splitting of the eluent unneeded (not published yet).

### 2.2. Interferences

Using conventional glass or Teflon^®^ PFA spray chambers and applying plasma conditions, resulting in an oxide formation of 2–5% oxide-based interferences on some of the investigated elements and, in particular, on the Pd isotopes do not allow the accurate quantification of these elements. Inefficient separation of Sr and Mo from the target analytes on the anion exchange resin resulted in a contribution of the respective oxide species, which were much higher compared to the low concentrations expected for the target analytes. We studied this effect by analyzing a standard curve containing 1–100-µg L^−1^ Mo in 0.024-M L^−1^ HCl (total Mo concentrations from 0.01 to 78.72 µg L^−1^ were measured in the investigated ground water samples; published in Reference [27] (the version in German language only)) with the matrix separation/pre-concentration method directly coupled to ICP-SFMS using a Scott-type Teflon^®^ PFA spray chamber. From the slopes of the resulting standard curves, a contribution of approximately 0.25% from Mo (as ^92^Mo^16^O^+^) on the ^108^Pd^+^ isotope was calculated. Considering the Pd concentrations in the samples being lower by at least a factor of 1000, this interference would have severe effects on the quantitative results. By using the APEX Ω membrane desolvation unit, the oxide formation rate, evaluated on the ^238^U^16^O^+^/^238^U^+^ ratio, could be reduced to ≤0.05%; hence, the oxide-based interferences were significantly reduced for all the elements prone to oxide formation. This was verified by the repeated measurement of a Mo calibration standard curve (0–100 µg L^−1^ in the eluent matrix) by flow injection using the SC *one*FAST six-port valve and a small sample loop (100-µL sample volume) instead of the matrix separation/pre-concentration system; a simulation of the “worst case”, viz., Mo, was quantitatively introduced into the ICP-MS (not only the fraction, which was retained on the anion exchange resin as the molybdate ion). The results did not show any detectable signal for ^108^Pd^+^; thus, the formation of oxide-based interferences could be sufficiently suppressed. The advantages of using a membrane desolvation unit could also be shown by Turretta et al. [31] for the direct determination of Pt in diluted seawater, which was hampered by the formation of ^179^Hf^16^O^+^ on ^195^Pt^+^.

However, mathematical interference correction of the isobarically interfering Cd isotopes on ^106^Pd^+^ and ^108^Pd^+^, referring to ^111^Cd^+^, was performed as described in Method 200.8, Revision 5.4 (1994) [32] to obtain accurate ^106^Pd/^108^Pd isotope ratios (the accuracy was evaluated based on the published values by IUPAC [33] for a natural abundance isotope standard and the certified abundances for the isotopically enriched standard). In 26 of the total 55 investigated ground water samples, total Cd concentrations of 0.004–0.47 µg L^−1^ were determined [27]. The contributing ^106^Cd (1.25% abundance) and ^108^Cd (0.89% abundance) signals were in the range of 1–33% (in two samples, even 258 and 378%) and 1–3% of the measured ^106^Pd and ^108^Pd isotopes, respectively. This approach was necessary, as uncorrected intensities, resulting in incorrect isotope ratios, would have increased the concentrations—depending on the Cd concentration—up to 27% and, in some samples, even by a factor of 2.5–8.3.

### 2.3. Matrix Effects

As already discussed earlier, the chloro complex species formation of the target analytes clearly depends on the chloride concentration in the sample, and the retention on the anion exchange resin depends on the fraction of negatively charged chloro complexes [13,29].

The total matrix content and composition (major ion concentration) may be important factors also, which influence the complexing characteristics and, consequently, retention on chelating sorbents and anion exchangers, respectively. As shown in Figure 1, the mineral waters investigated showed highly varying matrix compositions and concentrations (sum of the cations and anions), ranging from 26 mg L^−1^ to 7011 mg L^−1^. Approximately 30% of the samples showed major ion concentrations <100 mg L^−1^, which corresponds to the salinity of crystalline waters. Concentrations >1000 mg L^−1^ were measured in 12 samples; this can be attributed to saline aquifers. Thus, the effects of varying matrix compositions on the retention of Ag, Pd, Pt and Au were studied.

**Figure 6:**
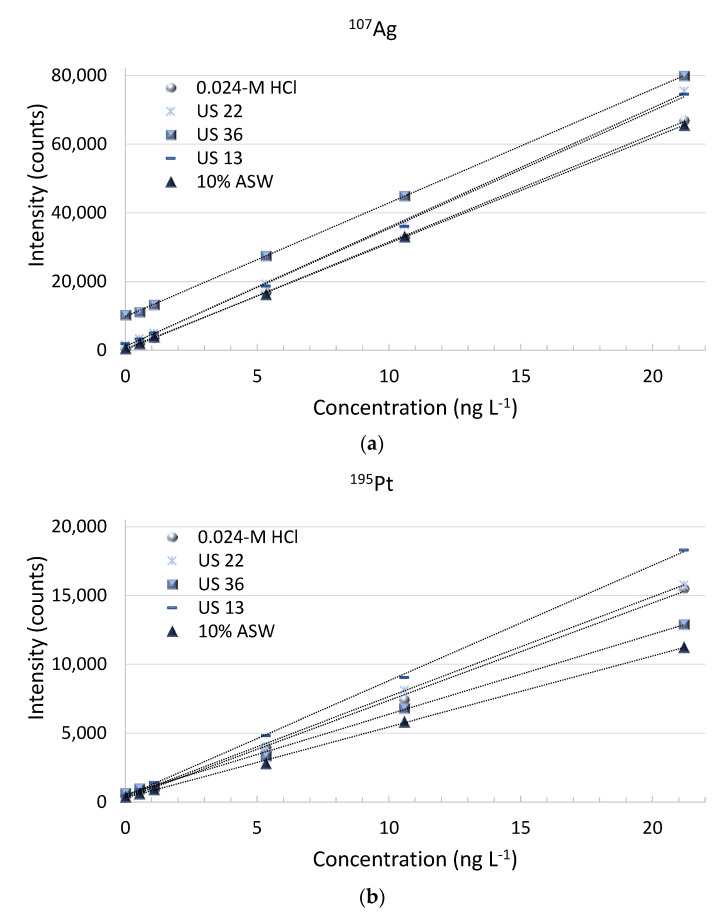
Calibration curves obtained for ^107^Ag (**a**) and ^195^Pt (**b**) in different matrices after matrix separation/pre-concentration.

Standard additions of concentrations ranging from 0 to 10 ng L^−1^ to the selected ground water samples (US 13: 6100 mg L^−1^, Cl^−^: 2004 mg L^−1^; US 22: 14.0 mg L^−1^; Cl^−^ <0.5 mg L^−1^; US 36: 1461 mg L^−1^, Cl^−^: 297 mg L^−1^) with varying characteristics (all acidified with 0.024-mol L^−1^ HCl) and an external calibration standard curve at 0.024-mol L^−1^ HCl were quantified. The results obtained are exemplarily shown for Ag and Pt (see Figure 6). As can be seen, the obtained sensitivities varied significantly. These results were expected due to the strong dependence of the chloride concentrations on the chloro complex formation—in particular, for Ag. An additional experiment was performed with samples with more similar characteristics (US 28, US 29 and US 31 with a maximum concentration of the major ions of 300 mg L^−1^ and <0.5–2.2-mg L^−1^ Cl^−^ concentration), and similar results were obtained. No clear trend, for instance, increasing/decreasing sensitivity with increasing/decreasing chloride concentrations and total major ion concentrations, respectively, could be observed. These results indicate that quantification by external calibration does not give accurate results, and therefore, an isotope dilution analysis is a prerequisite to compensate for this matrix effect. Due to the monoisotopic characteristics of Au, the application of standard additions had to be applied to obtain reasonable concentrations.

### 2.4. Validation

Validation parameters for the method, including instrument sensitivity, background equivalent concentrations (BEC) and detection and quantification limits (DL and QL), trueness and precision are summarized in the following tables. Table 2 shows the sensitivity of the method, determined from the slope of an external calibration in 0.024-M L^−1^ HCl and standard additions in seawater for comparison. As already discussed in Section 2.3, the matrix effects resulting from varying major ion compositions and concentrations were severe, leading to different slopes of the standard curves. The procedural blank concentrations (background equivalent concentrations—BEC, calculated based on the blank intensity and the intensity of a 1-ng L^−1^ standard in 0.024-M L^−1^ HCl), DL and QL, calculated as three and 10 times the standard deviation of the blanks (*n* ≥ 6) based on the sensitivity of the external calibration and from the blank concentrations of *n* = 8 ^108^Pd spiked blank samples, respectively, were given in ng L^−1^.

In Table 3, the quantification accuracy, based on a spike recovery experiment, is shown. Quantification was performed by IDA and matrix-matched external calibration for Au, respectively, on the native sample (*n* = 3) and the sample with a known concentration of the standard added. The recoveries were in the range of 97.4–119%.

Due to the lack of matrix reference materials certified for precious metals, we used the environmental matrix reference material TM 35 (Lake Ontario water), which is certified for Ag (3.7 µg ± 0.44 µg L^−1^), and repeatedly measured it using gravimetrical dilution by a factor of 500. The target value was 7.40 ± 0.88 ng L^−1^ and, hence, more in the range of the expected Ag concentrations in the investigated ground water samples. Quantification by IDA resulted in a mean Ag concentration of 7.46 ± 0.114 ng L^−1^, indicating trueness and high precision; however, in the diluted reference materials, Pd, Pt and Au could not be detected (see Table 4).

To estimate the quantification accuracy of Pd, Pt and Au, two Gabbro rock PGE reference materials (WGB-1 and WMG-1), certified for Pd, Pt and Au, were used after mineralization by microwave-assisted aqua regia/HF digestion and further dilution in 0.024-mol L^−1^ HCl. The approximate dilution factors, resulting from digestion and gravimetrical dilution of the digest, were 20,000 and 200,000, and the target values were calculated accordingly based on the certified values. Quantification was performed by external calibration in 0.024-mol L^−1^ HCl and IDA (Ag, Pd and Au) and IDA, respectively. The average concentrations ± 1 SD measured in the WGB-1 and WMG-1 reference samples were in agreement with the derived concentrations within the respective uncertainty, except for Pd in WMG-1, as the recoveries were 89% (external calibration) and 119% (IDA), respectively. Noticeably, the Pd- and Ag- concentrations (in WGB-1) quantified based on the slope of the external calibration curve were significantly lower than those measured by IDA; this might not be related to the matrix effects though, as the concentration of major constituents affecting sensitivity was regarded as negligible due to the high dilution in pure 0.024-mol L^−1^ HCl. The overall precision ranged from 0.5% to 9% relative standard deviation, but no relation could be seen with the type of quantification or values close to the QL.

The ratio precisions, estimated from the elution profiles of certified natural abundant standards for ^107^Ag/^109^Ag, ^106^Pd/^108^Pd and ^159^Pt/^196^Pt, are shown in Table 5. Short-term precision was calculated from *n* = 3 consecutive measurements and was in the range of 0.05%–0.66%, whereas that of Pd was significantly poorer than those obtained for Ag and Pt. Long-term precision was calculated from *n* = 20 measurements during a sequence of approximately 52 h. The ratio precisions increased up to 3.67%, and again, for Pd, the lowest precision was obtained. The mass bias per mass unit (MB) in percentage was calculated according to Xie and Kerrich [34] (MB–calculated as MB (%) = (R_*true*_/R*_measured_* − 1) * 100) considering the “true value”, as defined in the isotopic compositions of the elements from 1997, as defined by IUPAC [33]. The deviations from the “true value” were low (max—1.30%).

The repeatability of the measured isotope ratios of the natural abundance standards and of the measured isotope ratio of the enriched standards was taken into account for the calculation of the concentrations by the equation; hence, a correction factor *K* (*K* = measured ratio of the natural solution/natural abundance ratio) was not considered. Correction of the MB during the measurement sequence was performed by standard bracketing every sixth sample using the certified natural abundant standards; however, due to the low differences and the high precision, the impact on the result was negligible.

### 2.5. Background Concentrations in Ground Water

**Figure 7:**
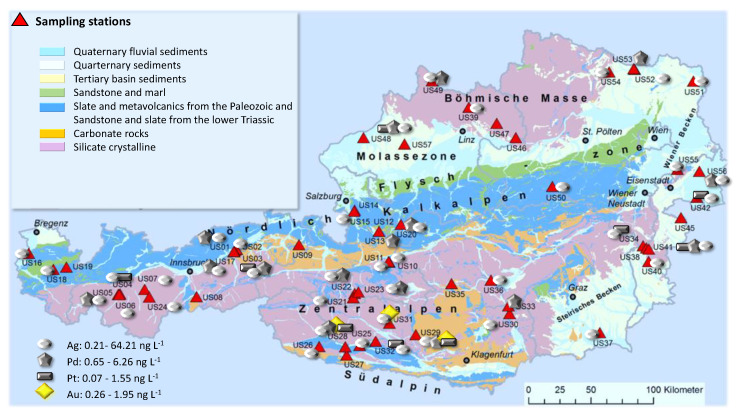
Sampling stations and occurrence of precious metals.

The novel methodological approach was applied to the analysis of the total concentrations of Ag, Pd, Pt and Au in a total of 55 mineral and medicinal waters for the evaluation of geogenic-derived background concentrations and potential anthropogenic emission. Table 6 represents the concentrations of these elements in the various samples, and in Figure 7, the sampling stations and occurrence of precious metals at the sampling sites are graphically depicted.

The mining sector has been an important economic factor since the medieval times, with a peak in the 16th century. Mining was not only focused on salt but, also, on iron and silver during this time. Austria was also rich in silver and gold deposits—in particular, in the Eastern Alps. Hence, it can be assumed that precious metals can also be found in ground waters due to the naturally occurring rock–water interactions.

Ag could be detected in all of the investigated ground water samples, and concentrations >QL ranged from 0.12 to 65.5 ng L^−1^, whereas the concentrations were <1 ng L^−1^ in about half of the samples. Significant amounts could be found in the samples collected in Tyrol in the region of the ancient silver mine Schwaz (US 02 and US 03) and in the Eastern Alps (in particular, in US 21 and US 29). As the occurrence of a variety of mineral deposits in the different geologic units of this region—in particular, in the Easter Tauern—is known, it is not surprising that traces of Pd, Pt and Au could also be measured. Au was quantified in three different samples originating from regions (US 28, US 29 and US 31) close to ancient gold mines—the center was Rauris (close to sampling stations US 21 and US 22)—but gold tunnels can still be found in the surrounding valleys of Salzburg, Carinthia and Tyrol. The Au concentrations ranged from 0.26 ng L^−1^ to 1.95 ng L^−1^. It is worth mentioning that the springs analyzed from these sampling stations also contained significant mean concentrations of As due to the occurrence of gold-bearing arsenopyrite. The Pd and Pt concentrations ranged from 0.32 to 6.34 ng L^−1^ and 0.22 to 1.11 ng L^−1^ and were measured in 34 and eight samples. There was no reference with respect to the occurrence of Pt/Pd mineral deposits in Austria, but it could be related to precious metal associations in the respective types of rocks and sediments. The concentrations measured were generally in the sub-ng L^−1^ to low ng L^−1^ range and represented natural (geogenic) background concentrations and ancient anthropogenic pollution, respectively. A more intense evaluation and interpretation of the data from a (hydro-) geochemical perspective was not within the scope of this study.

## 3. Materials and Methods

### 3.1. Chemicals and Laboratory Materials

High-purity nitric acid (HNO_3_; 65% of p.a. grade, Merck, Darmstadt, Germany) and hydrochloric acid (HCl; 37% of p.a. grade, Merck, Darmstadt, Germany) were prepared by double sub-boiling distillation with a duoPUR Quartz Sub-boiling system (MLS Lab Systems GmbH, Leutkirch, Germany). Due to a HCl/H_2_O azeotrope, the concentrated p.a. grade HCl (37%) was diluted with Milli-Q^®^ water to 10 mol L^−1^ prior to distillation to produce a consistent final concentration of 10-mol L^−1^ HCl. Milli-Q^®^ water (18.2 MΩ cm^−1^; SG Water GmbH, Barsbüttel, Germany) was single sub-boiling-distilled to obtain ultra-pure water.

A certified 1000-mg L^−1^ Ag single element ICP standard for trace analysis (Ag CertiPUR^®^, Merck, Darmstadt, Germany) and a certified multi-element standard (IV-Stock 28, Inorganic Ventures) containing 10 µg mL^−1^ each of Au, Hf, Ir, Pd, Pt, Rh, Ru, Sb, Sn and Te were used for the preparation of standards to perform external calibrations and standard additions, respectively. For the isotope dilution analysis (IDA), isotopically enriched standards (Ag enriched in ^109^Ag, abundance: 99.41% from ICS Science, Oviedo, Spain; Pt enriched in ^196^Pt, abundance: 97.25% and Pd enriched in ^108^Pd, abundance: 98.25%; both from the Science Technical Centre “Stable Isotopes” of State Scientific Centre of the Russian Federation–Institute of Physics and Power Engineering, Obninsk, Kaluga Region, Russia) were purchased.

Dowex^®^1x8 strong anion exchange resin in chloride form (trimethylbenzylammonium functional groups, 200–400 mesh, particle size: 34–74 µm) was purchased from Supelco (Bellefonte, PA, USA). TEVA^®^ Resin (also called Aliquat^®^ 336, quaternary ammonium functional groups, particle size: 50–100 μm) was kindly provided by TrisKEM International (Bruz, France).

The resins were precleaned by soaking an aliquot each in 5-mol L^−1^ double sub-boiled HNO_3_ in a 100-mL PE bottle for approximately 12 h while the slurry was shaken from time to time. Subsequently, the acid was decanted, and the resins were washed with portions of sub-boiled H_2_O until the decanted supernatant was at pH 5. PEEK columns (30 × 2.1-mm inner diameter; volume ≈ 104 µL, Supelco, Bellefonte, PA, USA) were filled with a slurry of precleaned Dowex^®^1x8 and TEVA^®^ Resin, respectively.

Before use, all materials involved in the sample collection, preparation and measurement processes (low-density polyethylene (LDPE) and polypropylene (PP) materials such as bottles used for water samples, vials, pipette tips, etc.) were acid leached with 10% and 1% (*v/v*) HNO_3_ 24 h each, rinsed three times with MQ water and dried on a Class 100 clean bench. Fluoropolymers (Teflon™ PFA (perfluoralkoxy) and FEP (fluoroethylene propylene) used for reagents involved in the matrix separation/pre-concentration process were cleaned with a traceCLEAN acid steam cleaning system (MLS Lab Systems GmbH, Leutkirch, Germany). Sample preparation and measurements were carried out under clean room conditions to avoid contaminations (Class 10,000 with metal-free clean benches Class 100, temperature: 20 °C and pressure: +5 Pa).

### 3.2. Samples and Reference Samples for Quality Control

Sample collection was carried out by the Geological Survey of Austria in the course of several sampling campaigns in September/October 2016 within the project “Mineral und Heilwässer Österreichs” (Mineral and Medicinal Waters of Austria) on behalf of the Austrian Federal Ministry of Agriculture, Regions and Tourism. The focus was on mineral waters according to regulation StF: BGBl. II Nr. 309/1999 [35] (no English version available) and state-approved mineral/healing springs with special characteristics regarding the mineral contents from all relevant geological fault zones of Austria’s territory. In total, 55 springs were selected for the analysis of Ag, Pd, Pt and Au. The samples were collected in 250-mL acid-cleaned PE bottles and acidified with double sub-boiled HCl (final concentration in the sample: 0.024 mol L^−1^) on-site. The samples were not filtered in order to determine the total concentrations of the metals. For quantification by IDA, the samples were spiked with the isotopically enriched standards at a final concentration of 10 ng L^−1^ of Ag, Pd and Pt. All sample bottles were double-packed in PE bags and stored at 4 °C until analysis.

Due to the lack of certified matrix reference materials for the target elements in natural waters at sub-µg L^−1^ concentrations, diluted TM-35 (trace element matrix reference material from Lake Ontario water), GEOTRACES SAFe North Pacific open ocean surface and deep water (SAFe S and SAFe D2) samples, as well as the certified reference materials WGB-1 and WMG-1 Gabbro Rock PGE Materials (Canadian Certified Reference Materials Project CCRMP, Ontario, Canada) after mineralization with aqua regia/hydrofluoric acid, were used to validate the method in terms of the trueness of the results.

### 3.3. Instrumentation

#### 3.3.1. Matrix Separation/Pre-Concentration

Analysis of the target elements Ag, Pd, Pt and Au was carried out using solid-phase extraction on the strong anion exchanger TEVA^®^ Resin, respectively, in a fully automated matrix separation/pre-concentration system prior to online coupling to ICP-SFMS.

**Figure 8:**
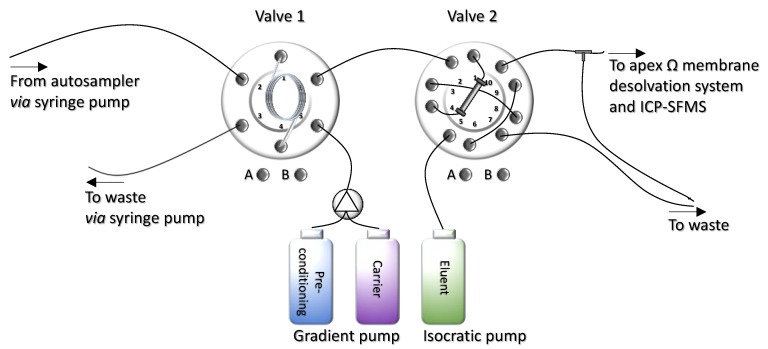
Schematic of the automated online matrix separation/pre-concentration flow injection system.

The set-up consisted of a metal-free high volume autosampler (AS-HV autosampler, Thermo Fisher Scientific) and a stepper motor-driven 10-mL syringe pump with a three-way distribution valve to aspirate and dispense measured quantities of liquid. The HPLC pump consisted of a dual pump system (ICS-3000, Thermo Fisher Scientific) with a gradient pump and an isocratic pump for transporting carrier and rinse solutions and elution acid. Moreover, a 6-port 2-postion valve and a 10-port 10-positon valve (configured to be used as a 6-port valve) (VICI^®^ Valco Instruments Co. Inc.) were used. Two sample loops made of PFA (4-mL volume each, 1-mm inner diameter; ESI Elemental Scientific Inc., Omaha, NE, USA) were connected to accomplish a total sample volume of 8 mL. All capillaries were made of PEEK (red: 0.125-mm inner diameter, green: 0.75-mm inner diameter and blue: 0.25-mm inner diameter). A PEEK three-port T piece (IDEX H&S P-715) was used as a flow splitter after the matrix separation/pre-concentration column (flow split 1:9, the flow was adjusted by connecting PEEK capillaries of different inner diameters to reduce the volume of elution acid entering the nebulizer to 100 µL). The matrix separation/pre-concentration system was controlled by HPLC software Chromeleon 6.80 (Thermo Fisher Scientific). Via two relays, the valves could be switched, and a third relay was used for triggering the ICP-SFMS for data acquisition. The scheme of the matrix separation/pre-concentration flow injection system is presented in Figure 8.

In the first step, the sample loop was loaded via the syringe pump operated in “pull mode” at a flow rate of 2 mL min^−1^. The loop was overfilled (total sample volume: 9.5 mL) to pre-rinse the loop, and excess volume was dispensed to waste. While the sample loop was loaded, the anion exchange resin was preconditioned with sub-boiled water. In the second step, valve 1 switched, and the sample was loaded onto the preconcentration column by the carrier at a flow rate of 2 mL min^−1^ (0.024-mol L^−1^ double sub-boiled HCl). The negatively charged chloride complexes were retained on the strong anion exchange resin, and unchelated matrix constituents were directed to waste. During this step, the sample uptake probe and the uptake capillary were rinsed with 0.2% (*v/v*) double sub-boiled HCl from the rinse port of the autosampler to prevent cross-contamination. In the third step, the preconcentration column was rinsed with the carrier solution at a flow rate of 2 mL min^−1^ for 2 min to remove the remaining sample and weakly bound ions. In the fourth step, the target analytes were eluted directly into the nebulizer of the membrane desolvation unit with 2-mol L^−1^ HNO_3_ and 0.5-mol L^−1^ HCl delivered by the isocratic pump of the IC in backflush mode for 5 min at a flow rate of 1 mL min^−1^ (flow split: 1:9) by switching valve 2. During elution, valve 1 switched to position A and back to B twice, thus cleaning the valve ports with the carrier. The automated sequence consisting of 4 steps required 15 min/sample.

#### 3.3.2. ICP-SFMS

The Element 2 High-Resolution ICP-SFMS (Thermo Fisher Scientific, Bremen, Germany) was used in low resolution mode for the determination of Ag, Pt, Pd and Au. In order to achieve the minimum oxide rates, calculated from the UO/U ratio, a membrane desolvation system (Apex Ω, ESI Elemental Scientific Inc., Omaha, NE, USA) consisting of a heated quartz cyclonic spray chamber and an EPTFE fluoropolymer membrane was used. The eluted analytes were introduced into the membrane desolvation unit through a microflow nebulizer (PFA MicroFlow Nebulizer, ESI). The Element 2 was equipped with a sapphire injector and nickel sampler and skimmer cones.

Before each measurement sequence, the tuning parameters were optimized in order to achieve the best sensitivity (approximately 4,000,000 cps for 1-µg L^−1^ In), lowest oxidation rate (<0.05% for UO/U) and high signal stability. The operation parameters are briefly summarized in Table 7, and the following isotopes were measured as transient signals: ^88^Sr, ^95^Mo, ^106^Pd, ^108^Pd, ^107^Ag, ^109^Ag, ^111^Cd, ^115^In (in the elution acid to monitor the signal stability), ^195^Pt, ^196^Pt and ^197^Au.

Data acquisition was performed in E-scan mode (495 runs * 1 passes, 10% mass window, 300 samples/peak and 1-ms sample time).

For integration of the measured transient signals, Chromeleon 6.80 (Thermo Fisher Scientific) was used.

### 3.4. Standardization

External calibrations with synthetic standards and matrix-matched external calibration standards, respectively, standard addition quantification and IDA, were tested for accurate quantification of the target elements in ground water. Synthetic calibration standards with known concentrations (0–10 ng L^−1^ of Ag, Pd, Pt and Au) were prepared in 0.024-mol L^−1^ HCl and processed for the samples. Matrix-matched external calibration standards were prepared in 1:10 diluted and undiluted seawater (acidified with 0.024-mol L^−1^ HCl) to represent a high(er) mineralization. Standard additions were performed on selected ground water samples. For the matrix-matched external calibration standards and the standard additions, a series of 6 standards containing Ag, Pd, Pt and Au with concentrations ranging from 10 to 1000 ng L^−1^ in 0.024-mol L^−1^ HCl were prepared. Each 100 µL of these standards were spiked to 10 g of the sample (10% seawater, undiluted seawater or ground water) to obtain the calibration curves.

IDA was used to quantify the concentrations of Ag, Pd and Pt in ground water. A working spike solution was prepared by serial gravimetrical dilution of a primary spike mixture in 0.024-mol L^−1^ HCl to obtain a final concentration of 1-µg L^−1^ Ag, Pd and Pt (=IDMS spike). Sample aliquots of 50 g were spiked with 0.5 g of the working spike solution. To determine the natural isotope ratios, natural abundance standards each containing 10-ng L^−1^ Ag, Pd and Pt were prepared gravimetrically from certified single (Ag) or multi-element standards (Pd and Pt) in 0.024-mol L^−1^ HCl. Accurate quantification of the working spike solution was achieved by applying reverse IDA, as described in a previous study [36]. The concentrations of Ag, Pd and Pt were calculated following the IDA equation as shown here:

$$c_{x}=c_{y}*\frac{m_{y}}{m_{x}}*\frac{\left(R_{y}-R_{b}\right)}{\left(R_{b}-R_{x}\right)}*\frac{f_{y}}{f_{x}}$$

*c_x_*: elemental concentration in the sample (mol g^−1^), *c_y_*: elemental concentration in the spike (mol g^−1^), *m_x_*: mass of sample solution in the blend (g), *m_y_*: mass of spike solution in the blend (g), *R_x_*: measured natural isotope ratio in a certified standard solution, *R_y_*: measured ratio of the spike, *R_b_*: isotope ratio measured in the blend, *f_x_*: constant abundance of the spike isotope in the sample and *f_y_*: certified abundance of the spike isotope in the spike solution. Since all ratios *R* were repetitively measured and the mass bias did not significantly change over the measurement sequence (validated from repetitive analysis of a natural isotope abundance standard), a mass bias correction factor was not applied. The ratios used for quantification were: ^107^Ag/^109^Ag, ^106^Pd/^108^Pd and ^195^Pt/^196^Pt.

## 4. Conclusions

We presented a fully automated online matrix separation/pre-concentration approach for the simultaneous determination of Ag, Pd, Pt and Au as their negatively charged chloro complexes in natural waters at ambient concentrations. The detection limits achieved were <0.1 ng L^−1^ and, thus, significantly lower than those obtained by previously reported methods with online sample treatments [26]. The use of membrane desolvation showed significant advantages for the reduction of oxide-based interferences. Moreover, it increased the signal intensities by a factor of 10 while not significantly influencing the noise. This resulted in great S/N ratios, which were essential, especially for the accurate analysis of Pd in the investigated water samples. Our approach opens the door to investigating naturally occurring concentrations of these elements at ultra-trace levels and anthropogenic influences in aquatic environments. This was demonstrated by successfully applying our method in a broad study to determine the geogenic background concentrations of Ag, Pd, Pt and Au in high-mineralized Austrian groundwater samples for the first time. The obtained dataset will serve as a base for future estimations of anthropogenic anomalies in aquatic environments.

## Figures and Tables

**Table 1:** Comparison of sensitivities (counts per ng L^−1^) and R^2^ obtained for Ag, Pd, Pt and Au in 1:10 diluted seawater after matrix separation/pre-concentration on Dowex^®^1x8 and TEVA^®^ Resin.

	Dowex^®^1x8		TEVA^®^ Resin	
	Slope	R^2^	Slope	R^2^
^107^Ag	1097	0.9978	5105	0.9972
^106^Pd	409	0.9958	1576	0.9978
^195^Pt	576	0.9941	2647	0.9971
^197^Au	1151	0.9983	2362	0.9982

**Table 2:** Analytical figures of merit for the method. Background equivalent concentrations (BEC) were calculated based on the blank intensity and the intensity of a 1-ng L^-1^ standard in 0.024-M L^−1^ HCl (*n* = 6). Detection and quantification limits (DL and QL) are defined as 3 and 10 times the standard deviation of the blank (*n* = 6) quantified by the slope obtained from the external calibration in 0.024-M L^−1^ HCl and from the isotope dilution analysis (IDA), respectively. Concentrations are given in ng L^−1^.

	Sensitivity (cps/ng L^−1^)			External Calibration		IDA	
	External Calibration	Standard Addition in Seawater	BEC (*n* = 6)	DL	QL	DL (*n* = 8)	QL (*n* = 8)
Ag (^107^Ag)	140,00	5100	0.022 ± 0.001	0.004	0.013	0.008	0.028
Pd (^106^Pd)	6900	2000	0.08 ± 0.028	0.045	0.15	0.092	0.31
Pt (^195^Pt)	8300	2600	0.021 ± 0.002	0.007	0.025	0.012	0.041
Au (^197^Au)	5800	2400	0.08 ± 0.012	0.026	0.088	-	-

**Table 3:** Quantification accuracy of a single standard addition to a ground water sample (US 04). Ag, Pd and Pt were measured by IDA, and Au was quantified by matrix-matched external calibration. Concentrations are ng L^−1^ and represent the average ± 1 SD.

	Unspiked Sample (ng L^−1^) (*n* = 3)	Spike Conc. Added (ng L^−1^)	Measured Average (ng L^−1^) (*n* = 2)	Recovery
Ag	2.10 ± 0.035	2.47	5.04 ± 0.050	119%
Pd	0.59 ± 0.018	2.31	3.24 ± 0.075	114%
Pt	0.39 ± 0.003	2.31	2.65 ± 0.066	97.4%
Au	<DL	2.31	2.37 ± 0.054	102%

**Table 4:** Trueness and precision for the reference samples (diluted TM 35 lot 0317 Lake Ontario water, certified for Ag, and diluted Gabbro Rock PGE reference materials WGB-1 and WMG-1). Concentrations are ng L^−1^ and represent the average ± 1 SD.

	External Calibration (ng L^−1^)	IDA (ng L^−1^)	Target Value (ng L^−1^)
TM 35 (1:500 diluted) (*n* = 6)			
Ag	n.d.	7.46 ± 0.114	7.40 ± 0.88
WGB-1 (1:20,000 diluted) (*n* = 3)			
Ag	2.31 ± 0.059	2.54 ± 0.052	Not certified
Pd	0.686 ± 0.0036	0.776 ± 0.015	0.757 ± 0.114
Pt	0.253 ± 0.005	0.269 ± 0.007	0.332 ± 0.087
Au	0.110 ± 0.0013		0.158 ± 0.060
WMG-1 (1:200,000 diluted) (*n* = 3)			
Ag	13.7 ± 1.24	13.6 ± 0.669	13.5 ± 1.49
Pd	1.69 ± 123	2.27 ± 0.072	1.91 ± 0.057
Pt	3.53 ± 0.158	3.59 ± 0.197	3.66 ± 0.183
Au	0.587 ± 0.020	-	0.550 ± 0.055

**Table 5:** Short- and long-term ratio precisions obtained from integrated peak areas of a series of elution profiles of certified natural abundant standards (10 ng L^−1^) and mass bias/mass unit considering the “true value”, as defined in the isotopic compositions of the elements (IUPAC)^33^.

		**RSD**	^**107**^ **Ag/** ^**109**^ **Ag Theoretical**	^**107**^ **Ag/** ^**109**^ **Ag Measured**	**Mass Bias/Mass Unit**
Short-term precision	R_x_ (*n* = 3)	0.07%	1.076	1.079	−0.27%
Long-term precision	R_x_ (*n* = 20)	0.95%	1.076	1.086	−0.87%
		**RSD**	^**106**^ **Pd/** ^**108**^ **Pd Theoretical**	^**106**^ **Pd/** ^**108**^ **Pd Measured**	**Mass Bias/Mass Unit**
Short-term precision	R_x_ (*n* = 3)	0.66%	1.033	1.024	0.87%
Long-term precision	R_x_ (*n* = 20)	3.67%	1.033	1.032	0.07%
		**RSD**	**^195^Pt/^196^Pt Theoretical**	**^195^Pt/^196^Pt Measured**	**Mass Bias/Mass Unit**
Short-term precision	R_x_ (*n* = 3)	0.05%	1.336	1.336	0.02%
Long-term precision	R_x_ (*n* = 20)	1.29%	1.336	1.354	−1.30%

Total measurement time: 51.5 h (for 3 days).

**Table 6:** Concentrations of Ag, Pd, Pt and Au in Austrian ground water samples analyzed by online ICP-SFMS after matrix separation/pre-concentration on the strong anion exchange resin TEVA^®^ Resin. Concentrations are in ng L^−1^.

	**US 01**	**US 02**	**US 03**	**US 04**	**US 05**	**US 06**	**US 07**	**US 08**	**US 09**	**US 10**	**US 11**	**US 12**	**US 13**	**US 14**	**US 15**	**US 16**	**US 17**		**US 18**	**US 19**	**US 20**
Ag	0.38	23.9	17.2	1.74	0.68	0.42	2.90	0.27	0.33	0.38	0.40	0.26	0.34	0.18	3.38	11.7	7.43		1.60	0.35	0.82
Pd	1.37	0.59	1.22	0.57	4.38	0.53	0.15	0.51	0.48	0.48	0.12	0.50	1.75	0.30	0.12	0.58	0.89		0.59	0.45	1.11
Pt	<DL	<DL	0.03	0.39	<DL	<DL	<DL	<DL	<DL	<DL	<DL	<DL	<DL	<DL	<DL	<DL	<DL		<DL	<DL	<DL
Au	n.d.	n.d.	n.d.	n.d.	n.d.	n.d.	n.d.	n.d.	n.d.	n.d.	n.d.	n.d.	n.d.	n.d.	n.d.	n.d.	n.d.		n.d.	n.d.	n.d.
	**US 21**	**US 22**	**US 23**	**US 24**	**US 25**	**US 26**	**US 27**	**US 28**	**US 29**	**US 30**	**US 31**	**US 32**	**US 33**	**US 34**	**US 35**	**US 36**	**US 37**		**US 38**	**US 39**	**US 40**
Ag	18.1	0.52	1.12	1.12	4.39	0.52	0.12	2.12	65.4	2.64	2.69	0.74	0.32	0.62	0.21	2.49	7.40		0.29	2.90	6.30
Pd	0.51	0.84	1.04	0.59	0.15	0.20	0.38	5.35	0.70	0.10	0.23	0.32	2.28	0.40	0.15	0.30	0.63		0.20	0.19	0.51
Pt	<DL	0.02	<DL	<DL	<DL	0.02	<DL	0.74	0.61	<DL	<DL	0.22	<DL	0.03	<DL	<DL	<DL		<DL	<DL	<DL
Au	n.d.	n.d.	n.d.	n.d.	n.d.	n.d.	n.d.	1.95	0.32	n.d.	0.26	n.d.	n.d.	n.d.	n.d.	n.d.	n.d.		n.d.	n.d.	n.d.
	**US 41**	**US 42**	**US 45**	**US 46**	**US 47**	**US 48**	**US 49**	**US 50**	**US 51**	**US 52**	**US 53**	**US 54**	**US 55**	**US 56**	**US 57**	**Min conc >QL**		**Max conc**		**DL**	**QL**
Ag	0.79	2.56	0.23	0.63	4.93	7.73	2.81	0.40	0.40	1.01	0.28	2.08	4.76	1.04	0.27	0.12		65.5		0.01	0.03
Pd	6.34	<DL	0.18	<DL	0.13	1.15	1.97	0.36	0.10	<DL	1.20	<DL	<DL	1.04	0.14	0.32		6.34		0.09	0.31
Pt	0.45	<DL	<DL	<DL	<DL	1.11	<DL	<DL	<DL	<DL	<DL	<DL	<DL	<DL	<DL	0.22		1.11		0.01	0.04
Au	n.d.	n.d.	n.d.	n.d.	n.d.	n.d.	n.d.	n.d.	n.d.	n.d.	n.d.	n.d.	n.d.	n.d.	n.d.	0.26		1.95		0.03	0.09

n.d.: not determined.

**Table 7:** Operating parameters of the apex Ω and ICP-SFMS.

Apex Ω Parameters	
Ar gas flow	0.3–0.32 mL min^−1^
N_2_ gas flow	0.7–0.8 mL min^−1^
Temperature	160 °C, 2 °C (spray chamber)
ICP-SFMS parameters	
Plasma power	1250 W
Nebulizer/Auxiliary gas flow	1.0 L min^−1^/0.8 L min^−1^
Cool gas flow	16 L min^−1^
Sample/skimmer cone	Nickel
Data acquisition	E-scan, 10% mass window, 30 ms
Masses monitored	LR (m/∆m = 300): ^88^Sr, ^95^Mo, ^105^Pd, ^106^Pd, ^108^Pd, ^107^Ag, ^109^Ag, ^111^Cd, ^115^In, ^195^Pt, ^196^Pt and ^197^Au

## Data Availability

Not applicable.
